# Bruch’s Membrane Compartmentalizes Complement Regulation in the Eye with Implications for Therapeutic Design in Age-Related Macular Degeneration

**DOI:** 10.3389/fimmu.2017.01778

**Published:** 2017-12-19

**Authors:** Simon J. Clark, Selina McHarg, Viranga Tilakaratna, Nicole Brace, Paul N. Bishop

**Affiliations:** ^1^Division of Evolution and Genomic Medicine, Faculty of Biology Medicine and Health, School of Biological Sciences, University of Manchester, Manchester, United Kingdom; ^2^Manchester Royal Eye Hospital, Manchester University NHS Foundation Trust, Manchester Academic Health Science Centre, Manchester, United Kingdom

**Keywords:** complement system proteins, age-related macular degeneration, Bruch’s membrane, diffusion, extracellular matrix, eye diseases

## Abstract

Age-related macular degeneration (AMD) is the leading cause of blindness in the western world and affects nearly 200 million people globally. Local inflammation driven by complement system dysregulation is currently a therapeutic target. Bruch’s membrane (BrM) is a sheet of extracellular matrix that separates the retina from the underlying choroid, a highly vascularized layer that supplies oxygen and nutrition to the outer retina. Here, we show that most complement proteins are unable to diffuse through BrM, although FHL-1, factor D and C5a can. AMD-associated lipid deposition in BrM decreases FHL-1 diffusion. We show that this impermeability of BrM creates two separate semi-independent compartments with respect to complement activation and regulation. Complement proteins synthesized locally on either side of BrM, or on the choroidal side if derived from the circulation, predominantly remain on their side of origin. As previous studies suggest that complement activation in AMD is confined to the choroidal side of BrM, we propose a model whereby complement activation in the choriocapillaris layer of the choroid generates C5a, which crosses BrM to interact with its specific receptor on RPE cells to initiate an inflammatory response in the retina. Understanding mechanisms underpinning AMD is essential for developing therapeutics that target the right molecule in the right anatomical compartment.

## Introduction

**Figure 1:**
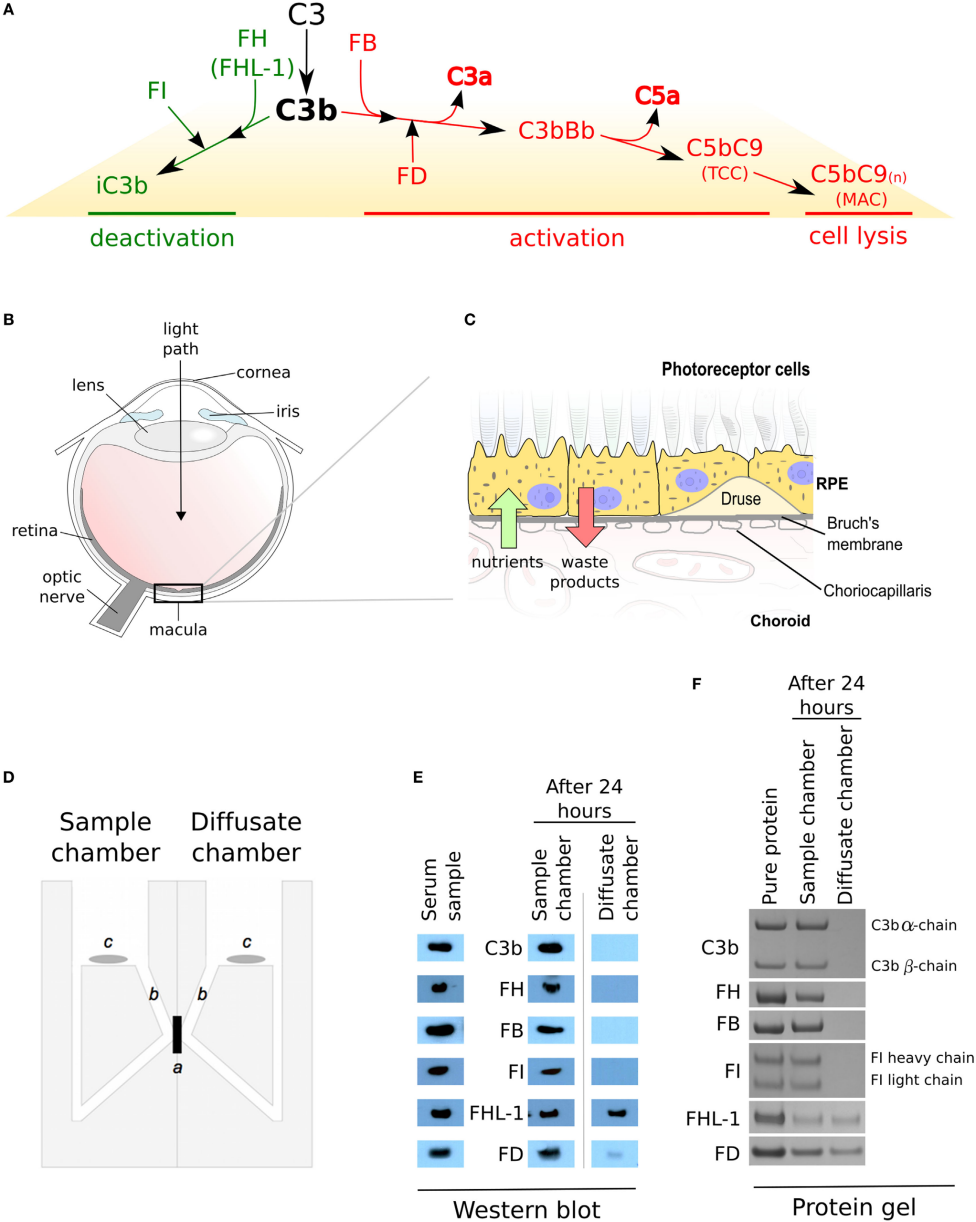
Complement regulation and selective diffusion of complement proteins across macular enriched Bruch’s membrane (BrM). **(A)** When C3b links onto surfaces, it either interacts with factor B (FB) and the complex is cleaved by factor D (FD) to form a C3 convertase (C3bBb) which feeds into the amplification loop, or it interacts with factor H (FH) (or FHL-1) which allows C3b cleavage by factor I (FI), thus preventing a complement response. Complement activation leads to increased release of the anaphylatoxins C3a and C5a, and results in the terminal complement complex which itself can become inserted into cell membranes (referred to as the membrane attack complex) causing cell lysis. **(B)** Schematic of the human eye highlighting the location of the macula. **(C)** BrM resides between the retinal pigment epithelium (RPE) cell layer and the choroid. Hallmark lesions of age-related macular degeneration, termed drusen along with diffuse lipid rich deposits called basal linear deposits, form within the BrM and disrupt nutrient flow from the choroid to the RPE cells. **(D)** An Ussing chamber was used in diffusion experiments, where *a*: enriched macula BrM; *b*: sampling access points; and *c*: magnetic stirrers, are shown. **(E)** Cropped images of western blots to detect soluble complement proteins in whole human serum and following diffusion through enriched BrM. Weaker band seen in the diffusate chamber for FD could imply an interaction with an unidentified serum partner that impedes its diffusion. Note that the antibodies used in these western blots only recognize one chain of either C3b or FI, thus only a single band is seen. **(F)** Cropped images of Instant Blue stained polyacrylamide gels following the introduction of purified complement proteins to the sample chamber, showing that the same complement proteins crossed enriched BrM as when serum was used in the experiments, but in this case, similar concentrations of FD were similar in the sample and diffusion chambers.

Genetic and tissue-based studies have implicated dysregulation of the complement system in age-related macular degeneration (AMD) (1–7). Most complement genes implicated in AMD are central components of the alternative pathway of complement activation, including *CFH, CFI, CFB*, and *C3* (3). Three distinct pathways activate the complement cascade including the classical, lectin, and alternative pathways, with all three converging on an amplification loop that if over-activated results inflammation and cell lysis (8). The cleavage of C3 into C3b and its deposition on surfaces [cells or extracellular matrix (ECM)] initiates the amplification loop, then complement factor B (FB) and factor D (FD) contribute to the formation of a C3-convertase (C3bBb), which drives forward the amplification loop by cleaving more C3 into C3b (Figure 1A). However, this cascade can be prevented through inactivation of C3b (forming iC3b) by the enzyme factor I (FI) and its cofactor, factor H (FH) or the truncated splice variant FHL-1, as iC3b cannot form C3-convertase. If these regulators fail to control the complement cascade, downstream consequences include the release of the anaphylatoxins C3a and C5a, and ultimately the production of the terminal complement complex (TCC: referred to as the membrane attack complex (MAC) when it becomes integrated into cell membranes causing lysis and death). So the deposition of TCC/MAC can be regarded as a marker of prior complement activation.

In AMD, the damage is largely concentrated within the central part of the retina, i.e., the macula (Figure 1B). The retina consists of the neurosensory retina (which contains the photoreceptor cells amongst others) and the retinal pigment epithelium (RPE) (Figure 1C). Underneath the RPE is a highly vascularized layer called the choroid; the inner part of the choroid, the choriocapillaris, contains fenestrated capillaries that are separated from the RPE by a sheet of ECM called Bruch’s membrane (BrM). Complement proteins that reach the macula and underlying choroid could be derived systemically from the choroidal circulation, as the main site of synthesis is the liver, but there is also evidence for local production by the RPE, neurosensory retina and within the choroid. There is ample evidence that the RPE BrM/choriocapillaris complex is involved in AMD pathogenesis, but immunohistochemical studies of TCC/MAC deposition in human eye tissue provide evidence that the main site of complement activation is the choriocapillaris layer and not the RPE (2, 7, 9, 10), suggesting that BrM acts as a barrier to complement proteins and therefore complement activation. Here, we show that while most complement proteins cannot cross BrM, specific complement proteins can cross this barrier. We propose a model whereby there are two semi-independent compartments with regard to complement regulation, but the ability of specific complement components to cross BrM plays an important role in AMD pathogenesis.

## Materials and Methods

### Reagents

Whole human serum used in the diffusion experiments was purchased from Sigma-Aldrich (Poole, UK, catalog no. H4522) as was purified human IgG (Sigma-Aldrich, catalog no. I2511). Commercial antibodies used include: from R&D Systems (Abingdon, UK) anti-FI (clone 271203, catalog no. MAB3307); anti-FD (clone 255706, catalog no. MAB1824); and anti-FB (clone 313011, catalog no. MAB2739): from Abcam (Cambridge, UK) anti-FH (clone OX23, catalog no. ab17928): and Cambridge Biosciences (Cambridge, UK) anti C3b (clone 755, catalog no. 2072). An in-house polyclonal antibody specific for FHL-1 was used and has been described and characterized previously (11). Commercially available purified complement proteins used include C3b (VWR International, Lutterworth, UK, catalog no. 204860), C3a (R&D Systems, catalog no. 3677-C3), C5a (R&D Systems, catalog no. 2037-C5), FH (Sigma-Aldrich, catalog no. C5813), FB (VWR International, catalog no. 341262), FI (VWR International, catalog no. 341280), and FD (VWR International, catalog no. 341273). The FHL-1 protein (both 402Y and AMD-associated 402H forms) was made in-house using a method described previously (11). Molecular weight markers used in SDS-gels were Blue Prestained Protein Standards, Broad Range (11–190 kDa, New England BioLabs, Hitchin, UK, catalog no. P7706S). Deglycosylation of complement proteins was undertaken using Remove-iT PNGase F (New England Biolabs, catalog no. P0706S) and magnetic chitin beads (New England Biolabs, catalog no. E8036S).

### Human Donor Eye Tissue

Details of donor eye tissue used in this study are listed in Table 1. Human eyes were obtained from the Manchester Royal Eye Hospital Eye Bank after removal of the corneas for transplantation. Our research adhered to the tenets of the Declaration of Helsinki. In all cases, there was prior consent for the eye tissue to be used for research, and guidelines established in the Human Tissue Act of 2004 (UK) were followed. Ethical approval for the use of human donor eyes was given by the University of Manchester Local Research Ethics Committee 3 (ref. 11305).

**Table 1:** List of donors used in this study.

Donor number	Sex	Age	Ave. age	Experiment
1	F	70	74	Whole serum diffusion
2	F	76		
3	M	81		
4	F	63		
5	F	81		

6	M	73	73	Purified protein diffusion—old
7	M	75		
8	F	63		
9	M	77		
10	F	79		

11	M	41	32	Purified protein diffusion—young (inc. factor I function tests)
12	M	29		
13	M	20		
14	F	37		
15	M	35		

16	F	76	74	Deglycosylated diffusion
17	M	75		
18	M	69		
19	F	75		
20	M	76		

21	F	91	87	Age-related macular degeneration (AMD) samples
22	F	85		
23	M	94		
24	F	76		
25	M	91		

26	M	82	85	Non-AMD ctrls.
27	F	88		
28	F	84		
29	F	82		
30	F	89		

31	M	69	73	Whole IgG and FAB fragment diffusion
32	M	68		
33	M	66		
34	M	83		
35	F	77		

36	F	86	73	C3a and C5a diffusion
37	F	58		
38	M	71		
39	M	85		
40	M	66		

### Ussing Chamber Diffusion Experiments

The passive diffusion of soluble proteins through enriched macula BrM was investigated as described previously (11). Briefly, the macular region of enriched BrM (12) isolated from donor eyes (Table 1) was mounted in an Ussing chamber (Harvard Apparatus, Hamden, USA); except where specified, eye tissue that the BrM was removed from did not show macroscopic evidence of AMD or other macular pathology. Once mounted, the 5 mm diameter macular BrM was the only barrier between two identical compartments (see Figure 1D). Both sides of BrM were washed with 2 ml of PBS for 5 min at room temperature. Human serum was diluted 1:1 with PBS and 2 ml was added to the Ussing compartment designated the sample chamber. Alternatively, purified proteins were added to the sample chamber in PBS at 100 µg/ml. After 1 min, if no leaks were detected into the second compartment (which would indicate a compromise in membrane integrity), 2 ml PBS alone was added to the second compartment of the Ussing chamber, designated the diffusate chamber, and it was then left at room temperature for 24 h with gentle stirring in each compartment to avoid generating gradients of diffusing proteins. Samples from each chamber (20 µl) were analyzed by gel electrophoresis. Pre-cast 4–12% NuPAGE Bis Tris SDS gels (Thermo Fisher Scientific, Altrincham, UK) were run for 60 min at 200 V under reducing conditions. Gels were either stained with Instant Blue stain (Expedeon, Harston, UK) for 60 min at room temperature or subject to Western blotting. In order to calculate the percentage of protein in the sample or diffusate chambers, band densities in the Instant Blue stained SDS gels were measured using ImageJ64 (version 1.40g; http://rsb.info.nih.gov/ij). The average intensity of these bands, over five separate experiments, were compared to the density of control bands that represent 100% loaded protein (i.e., 20 µl of 100 µg/ml). The calculated percentage protein was then plotted ± SD.

For western blotting, a semi-dry transfer apparatus was used applying 80 mA for 1.5 h, and the proteins were transferred onto the nitrocellulose membrane in a buffer containing 25 mM Tris, 192 mM glycine, 10% (v/v) methanol. Membranes were blocked in PBS, 10% (w/v) milk, 0.2% (w/v) BSA for 16 h at 4°C before addition of antibodies against complement proteins at 0.5 µg/ml, in PBS, 0.2% (v/v) Tween-20 (PBS-T) for 2 h at room temperature. Membranes were washed 2 × 30 min in PBS-T before the addition of a 1:2,000 dilution of HRP-conjugated secondary antibody for 2 h at room temperature. Membranes were washed 2 × 30 min in PBS-T before the addition of SuperSignal West Pico Chemiluminescent Substrate (Thermo Fisher Scientific, catalog no. 34080) for 3 min at room temperature. Reactive bands were detected by exposing Super RX-N X-ray film (FujiFilm, catalog no. PPB5080) to the treated membrane for 5 min at room temperature and developed on an automated X-ray film developer.

### Oil Red O Staining of Excised Enriched Macula BrM

Enriched macular BrM used in diffusion experiments was excised from the Ussing chamber, embedded in OCT cryoprotectant and frozen at −80^o^C prior to cutting 10 µm frozen sections using a cryostat. These were stained for esterified cholesterol and other lipids with the lipid marker Oil Red O following previously published protocols (13). Briefly, the frozen sections were fixed with 4% paraformaldehyde for 15 min at room temperature before washing with deionized water followed by 60% (v/v) isopropanol. Tissue sections were then treated for 40 min at room temperature with freshly prepared 0.3% Oil Red O in isopropanol prior to washing in water and applying cover slips in mounting medium. To confirm specificity of Oil Red O staining, some samples were pre-treated with 100% acetone at room temperature for 10 min in order to remove lipids. Acetone treated tissue were then washed three times for 5 min each in distilled water prior to staining with Oil red O. Imaging was performed using a Pannoramic 250 Flash II slide scanner system (3DHistech, Budapest, Hungary). Tissue sections from rat aorta were used as a positive control for Oil Red O staining.

### C3b Breakdown Assay

The C3b breakdown assays used in this work to test the functional capacity of FI were carried out as described previously (11). Briefly, reactions were conducted in a total volume of 20 µl. 2 µg purified C3b and 0.1 µg FHL-1 were mixed together in PBS, and 0.04 µg either purified FI, deglycosylated FI (dFI), or a 10 µl sample taken from the diffusate chamber of a diffusion experiments were added and left to incubate at 37°C for 15 min. Reactions were stopped with the addition of 5 µl 5× SDS reducing sample buffer and boiling at 100°C for 10 min. Samples were analyzed by SDS-PAGE and stained with Instant Blue as described above and the density of the 68 kDa iC3b product band was measured using ImageJ64 (version 1.40g; http://rsb.info.nih.gov/ij) and used to track C3b breakdown efficiency. In each case, averaged data from three separate experiments were used and data points are plotted ± SD.

### Deglycosylation of Complement Proteins

Remove-iT PNGase F, which is tagged with a chitin-binding domain, was used to deglycosylate (by removing N-glycans) purified C3b, FH, FB, and FI proteins under non-denaturing conditions. In each case, 20 µg protein, in a total volume of 18 µl, was used to which 2 µl of GlycoBuffer 2 (10×) was added. After gentle mixing by aspiration, 5 µl of the supplied PNGase F was added and carefully mixed by aspiration. A control sample of FI was subjected to the same conditions except that the PNGase F was replaced with PBS, this was used to ensure that the protocol conditions did not alter FI function. Reactions were left in a water bath at 37°C for 24 h. For the subsequent removal of PNGase F, 50 µl of magnetic chitin beads were washed in PBS, added to the deglycosylation reaction and incubated at room temperature for 10 min. Magnetic chitin beads and associated PNGase F were pelleted using a magnetic stand and the supernatant containing the deglycosylated protein collected.

### Measurement of Protein Dynamic Radius by Dynamic Light Scattering

DLS measurements were performed with Zetasizer Nano-S (Malvern, Herfordshire, UK) at a controlled temperature of 25°C. Samples were diluted in PBS at a concentration of 1 mg/ml and then spun at 14k RPM for 15 min before being loaded into a 45 µl quartz cell. Scattering intensity fluctuations at 90° were collected averaging 14 readings and three replicates. Scattering was correlated over time and the mean particle size (Z-average) fitted by exponential regression within the Zetasizer software. The volume distribution of the particles was converted using Mie theory to generate a more realistic interpretation of the particle distribution within the sample.

## Results

### Selective Diffusion of Complement Proteins across BrM

The diffusion of complement proteins involved in the alternative pathway through enriched BrM from human donor eyes (12) (see Table 1 for donor details) was investigated using an Ussing chamber (Figure 1D). The ability of proteins to cross BrM was assessed by western blotting aliquots from both the sample and diffusate chambers after 24 h. We found that FD, and as we have observed previously, FHL-1 (11), diffused through BrM, whereas C3/C3b, FH, FB, and FI did not (Figure 1E). To exclude the possibility that C3/C3b, FH, FB, and FI did not diffuse through the enriched BrM because of interactions with other serum components, we repeated the experiments with purified complement proteins, and after 24 h obtained the same results (Figure 1F); therefore, these diffusion properties are intrinsic to the complement proteins themselves and not conferred upon them by external interactions in sera. By western blot FD diffusion appeared partial over the 24-h period of the experiment, as the diffusate and serum compartments did not appear to contain the same concentration of FD. However, when purified FD was used the concentrations in the two compartments were similar; a possible explanation is that FD did interact with another serum component. To investigate if age was a factor in the observed selective diffusion pattern, we used the purified proteins and tested diffusion across enriched BrM from five younger donors (ages 20–41) and five older donors (ages 63–79), see Table 1 for details, but the same pattern was observed showing that donor age did not affect diffusion.

**Figure 2:**
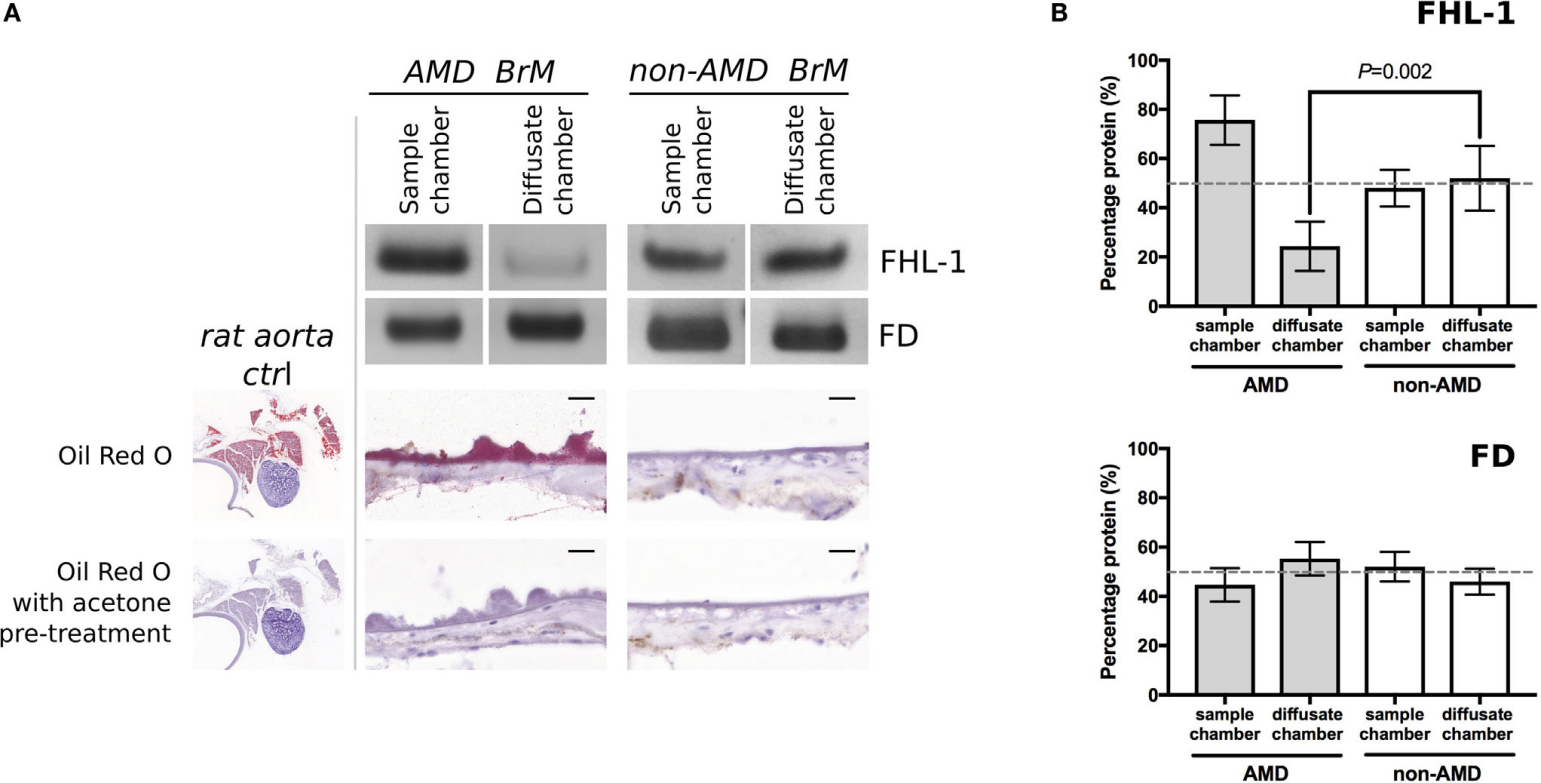
Increased lipid deposition in samples from donors with early age-related macular degeneration (AMD) perturbs diffusion through macular enriched Bruch’s membrane (BrM) of FHL-1, but not factor D (FD). **(A)** Cropped images of Instant Blue stained polyacrylamide gels showing that FHL-1 is not able to diffuse as freely through enriched BrM from donors with early AMD (as defined by presence of drusen) compared to non-AMD donors, whereas the diffusion of FD is unhindered. Frozen sections of AMD and non-AMD enriched BrM stained with Oil Red O showing lipid deposition and drusen in the AMD affected enriched BrM. Frozen sections of rat aorta were used as a positive control for Red Oil O staining. Scale bars 20 µm. **(B)** Percentage of protein present in both Ussing chamber compartments showed that, while FD reaches equilibrium across AMD macular BrM, only an average of 25% FHL-1 crossed BrM (over five separate experiments, error bars represent ± SD).

Next, we investigated whether FHL-1 and FD could still diffuse across enriched macula BrM from donors with early AMD. We found that diffusion of FHL-1 (both the 402Y and AMD-associated 402H variants) through enriched BrM with early AMD was perturbed (Figure 2A): around 75% of added protein was retained in the sample chamber. FD diffusion continued to reach equilibrium unabated (Figure 2B). To confirm the AMD status of these samples, the tissue was stained using Oil Red O, which demonstrated the increased levels of lipid deposition in the BrM and large drusen associated with AMD (13, 14).

### Glycosylation of FI Prevents Its Diffusion through BrM

**Figure 3:**
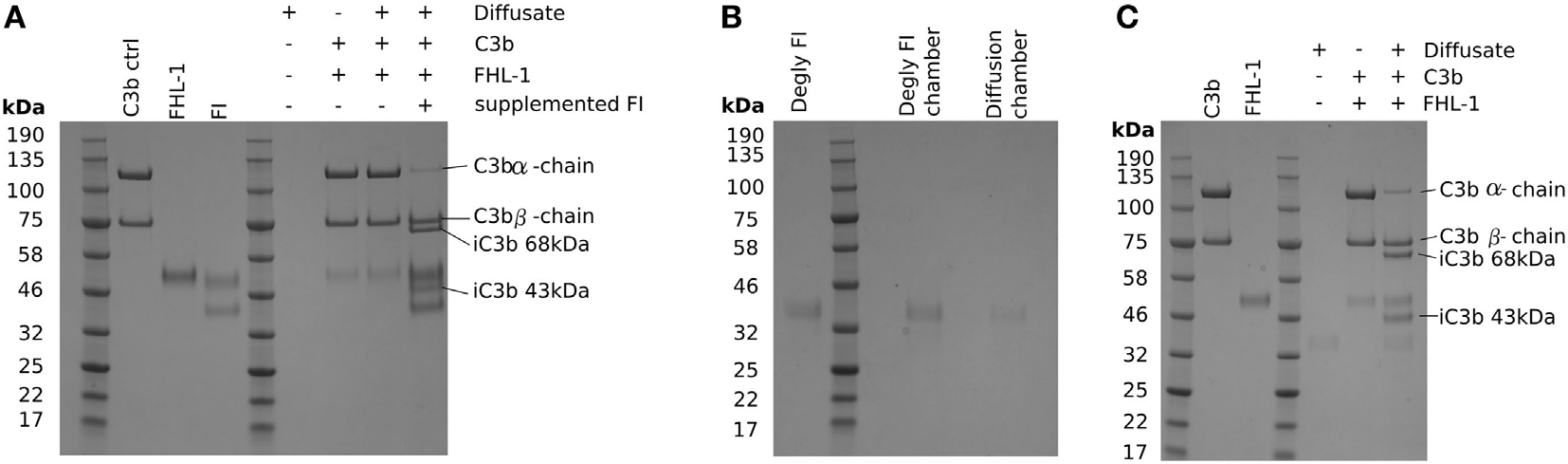
Deglycosylation of factor I (FI) allows it to diffuse through Bruch’s membrane (BrM). SDS-PAGE gels stained with Instant Blue: **(A)** showing that glycosylated pure FI is not able to pass through BrM into the diffusate chamber and confer C3b breakdown. The assay validity was checked by the supplementation of pure FI to induce C3b breakdown, **(B)** deglycosylation of FI causes allows the protein to diffuse through BrM, **(C)** the deglycosylated FI present in the diffusate chamber is able to confer complement regulation by mediating C3b breakdown.

The protein molecular weight “cut-off” of BrM for diffusion has been investigated previously (15, 16), and it was estimated that there was selectivity in protein diffusion in the molecular weight range of 100–200 kDa, which decreases with age: little apparent selectivity was observed below 100 kDa (15). However, even though the molecular weight of the FI core protein is 65 kDa, it did not diffuse through BrM. Given that only small quantities of FI are required to cleave C3b, we investigated the possibility that FI could pass through BrM, but at a level below that we could detect directly. To achieve this, we employed a C3b breakdown assay, where it is possible to observe the breakdown of the α-chain of C3b in the presence of FHL-1 and FI. Addition of FHL-1 and C3b to an aliquot from the diffusate chamber sample demonstrated a lack of C3b α-chain breakdown, i.e., an absence of FI (Figure 3A). Assay validity was confirmed by the addition of purified FI directly to the same aliquot and the subsequent degradation of the C3b α-chain into its constituent 43 and 68 kDa iC3b breakdown products (Figure 3A). FI is a glycoprotein and is substituted with N-glycans, as indeed are many complement proteins and their function can be modified by glycosylation (17). We hypothesized that the glycosylation added significantly to the hydrodynamic size of FI and thereby prevented it from crossing BrM: neither FHL-1 or FD is glycosylated (17, 18). Therefore, we deglycosylated FI (dFI: Figure S1 in Supplementary Material) and when used in the Ussing chamber found that the dFI showed increased diffusion capacity where, on average, 20% of dFI appeared in the diffusate chamber after 24 h (Figure 3B). The dFI material that crossed the enriched BrM was found to be functionally active by its capacity to cleave C3b into iC3b in the presence of FHL-1 (Figure 3C). Other complement proteins that did not cross the enriched BrM were also glycosylated, so in further experiments these were deglycosylated. However, deglycosylated FH and FB still did not cross the enriched BrM; it was not possible to deglycosylate C3/C3b in a non-reducing manner so we could not perform the experiment for C3/C3b (Figure S2 in Supplementary Material).

### Diffusion of the Anaphylatoxins C3a and C5a through BrM

**Figure 4:**
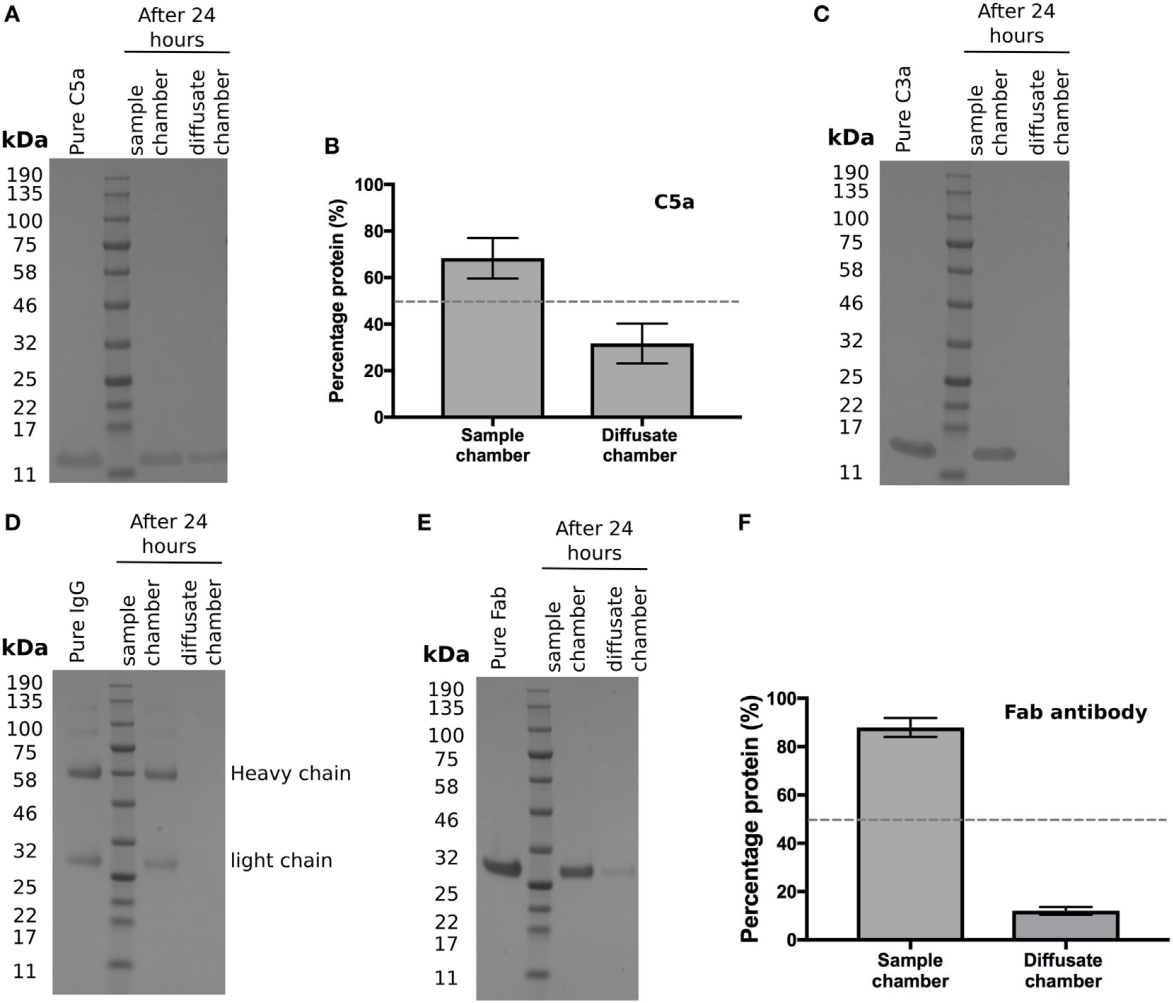
Selective permeability of Bruch’s membrane (BrM) to complement analphylatoxins and antibodies **(A,B)** Purified C5a diffused across enriched BrM over 24 h, while **(C)** no purified C3a was detected in the diffusate chamber after 24 h. **(D)** Whole IgG did not cross enriched BrM samples, whereas **(E)** the Fab antibody fragment ranibizumab did cross enriched BrM, but did not reach equilibrium after 24 h, where only an average of ~12% of the Fab antibody was detected in the diffusate chamber **(F)**. Data shown are representative of five separate experiments; error bars in **(B)** and **(F)** represent ± SD.

Activation of the complement cascade releases the analphylatoxins C3a and C5a; these are both pro-inflammatory and stimulate vascular endothelial growth factor (VEGF) transcription (19–21). As they are small (<10 kDa), proteins we investigated whether they could cross BrM using the Ussing chamber. After 24 h, recombinant C5a was detected in the diffusate chamber (Figure 4A), with an average of 32 ± 5% of the total protein added crossing BrM (Figure 4B). Despite its small size, no detectable levels of recombinant C3a crossed the enriched macula BrM in our test system (Figure 4C).

### Diffusion of Antibody Derived Therapeutics through BrM

The above data suggests that BrM compartmentalizes the regulation and hence activation of complement. Therefore, whether delivered by intravitreal injection or systemically it is relevant to know whether antibody-based therapeutics could cross BrM to reach their target. With an ever-increasing interest in the development of antibody therapies derived against complement proteins, we tested to see if whole IgG could diffuse through BrM but were unable to detect any IgG in the diffusate chamber after 24 h (Figure 4D). Next, we determined whether a Fab fragment antibody could cross BrM (using Ranibizumab as it was readily available to us) (Figure 4E). Ranibizumab was detected in the diffusate chamber after 24 h, although given its molecular weight (of ~47kDa) and lack of glycosylation, a surprisingly small amount of was detected (on average ~12% of added protein) (Figure 4F).

## Discussion

Here, we investigated the permeability of macular BrM to complement proteins. BrM is a thin (2–4 µm) sheet of ECM composed of a central elastic layer flanked by inner and outer collagenous layers. External to the collagenous layers are the RPE basement membrane on one side and choriocapillaris basement membrane on the other (14). As described previously (12), it is not possible to entirely remove the choriocapillaris layer from BrM as they share a basement membrane and the intercapillary septa (also called pillars, which are situated between the capillaries) merge with BrM; therefore, we refer here to the samples we used as enriched BrM. Moore and Clover (15) demonstrated that proteins up to 100 kDa can pass across BrM and larger proteins to a variable extent (15). Furthermore, they demonstrated a decline in permeability with aging. While such studies provide a broad overview, individual proteins need to be assessed, as it is clear their hydrodynamic size, shape, and post-translational modification (especially glycosylation), rather than just simply their molecular weight, determines mobility across BrM, along with their propensity to interact with BrM components (see Table 2). Of particular interest is the differing mobility of C3a versus C5a across BrM (Figures 4A–C). Both anaphylatoxins are small in molecular weight and share almost identical hydrodynamic radii, but C3a is calculated to have a significantly greater net positive charge that C5a (Table 2). Given BrM is loaded with negatively charged glycosaminoglycans [such as heparan sulfate (22, 23)], it is plausible that C3a becomes bound and collects on/within BrM.

**Table 2:** Summary table of complement proteins used in experiments.

Protein	MW (KDa)^a^	Diffusion		Glycosylation	Net charge^b^ (pH7)	Dynamic radius (nm)
		>70 years old	<35 years old			
C3	180	No	No	Yes	−11.8	4.84
Factor H	155	No	No	Yes	−9.6	5.56
Factor B	83	No	No	Yes	0.7	3.22
Factor I	65	No	No	Yes	2.7	3.07
FHL-1	49	Yes	Yes	No	−1.5	4.40
Factor D	24	Yes	Yes	No	1.1	2.08
C3a	9	No	–	No	9.2	1.56
C5a	8.3	Yes	–	Yes	5.2	1.63

*^a^MW values are based on core protein and do not include the contribution of glycosylation*.

*^b^Net charge calculated using protparam v3.4 tool from Expasy (https://web.expasy.org/protparam/)*.

The data presented here suggests that the choroid and retina can regarded as semi-independent as far as complement regulation is concerned, as they are partitioned by BrM and have different sources of complement proteins. Circulating complement proteins and local production within the choroid itself could supply the choroid. It should be noted that despite the fact that the choriocapillaris is fenestrated, it does not allow the free transport of large macromolecules out of the circulation, rather this is regulated by cellular processes including caveolae-mediated transcytosis (24); so, the endothelial cells of the choriocapillaris will regulate the egress of complement proteins from the circulation. Because of the barrier properties of BrM, the outer retina will largely rely on local complement protein production. In addition, the RPE could supply complement proteins to BrM and the choriocapillaris layer. In particular, we have previously shown that FHL-1 is expressed by the RPE and is the main complement regulator in BrM and the intercapillary septa of the choriocapillaris layer. If this FHL-1 is supplied by the RPE then the accumulation of drusen and lipid in BrM could disrupt transport and make these tissues more susceptible to complement activation. It is also of note that proteins that are secreted basolaterally by the RPE, if they are unable to diffuse through BrM, may accumulate at the RPE/BrM interface with detrimental consequences.

**Figure 5:**
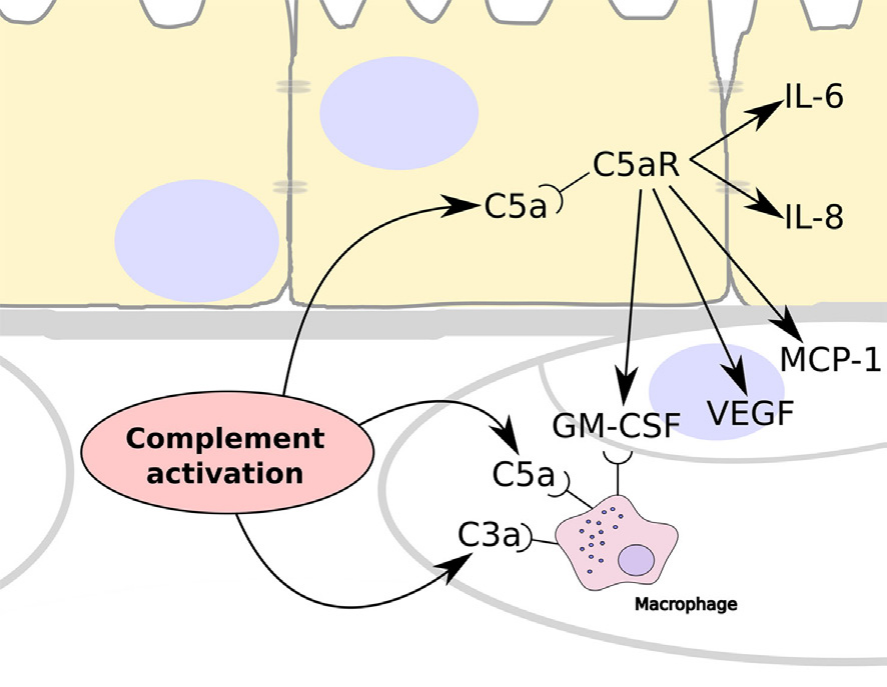
Summary schematic showing the consequences to complement activation in the choriocapillaris layer of the choroid. Over-activation of the complement system leads to the generation of C3a and C5a, which are able to recruit and stimulate circulating macrophage in the choroid. In addition, C5a can cross Bruch’s membrane and stimulate RPE cells *via* their C5a receptors (C5aR). This results in the release of IL-6 and IL-8 by the RPE cells, and the expression of vascular endothelial growth factor (VEGF), monocyte chemoattractant protein-1, and granulocyte macrophage colony-stimulating factor (GM-CSF), which stimulates angiogenesis and the recruitment of immune cells such as macrophages.

It has been shown that TCC/MAC is deposited in the choriocapillaris, while there is very little deposition in the retina, and there is increased evidence of this deposition in eyes from donors that had AMD or had a genetic predisposition to AMD (2, 25). This indicates that complement activation occurs primarily in the choriocapillaris layer. Complement activation releases C5a and we propose that because this can cross BrM it induces inflammation by binding to its receptor, C5aR, which is expressed on RPE cells (26) and stimulate their production of VEGF (19–21) (Figure 5). In addition, C5a stimulation of RPE cells could induce the expression of monocyte chemoattractant protein-1 and granulocyte macrophage colony-stimulating factor, promoting the recruitment of immune cells such as macrophages as has been demonstrated in AMD (27, 28). The lack of ability of C3a to pass through BrM coincides with a lack of expression of the C3a receptor (C3aR) by the RPE (26).

There has been recent interest in the use of intravitreally delivered purified complement regulators (such as FH or variations thereof) as a method for re-addressing complement over activation (29, 30), but our results suggest greater care must be taken over their selection and design, e.g., purified FH, will not penetrate the BrM and therefore reach the site of complement over activation associated with disease. There is also an interest in intravitreally delivered antibody therapeutics to suppress complement activation and thereby treat geographic atrophy, a form of advanced AMD. One such therapeutic is lampalizumab, an anti-FD Fab fragment which is currently undergoing two phase III clinical trials for geographic atrophy (NCT02247479 and NCT02247531) after positive outcomes in a phase II trial (31). Our study suggests that it has potential to be effective being a Fab fragment that can cross BrM and thereby target FD on both sides of BrM, although it is unknown how long it would remain in the choroid. However, very recently it has been reported that one of the trials failed to reach its 1-year milestone of slowing the progression of geographic atrophy. This, along with other failures of complement therapeutics for patients with geographic atrophy suggests that it would be more effective to target complement activation earlier in the disease process. It is probable that complement over-activation precedes the RPE cell loss and atrophy of the choriocapillaris that characterize geographic atrophy and that by the time these interventions are being given other damaging biochemical pathways have been activated that have become independent from the original complement over-activation (32). So despite the benefits of using geographic atrophy-based clinical trial models, for best results, it may be desirable to administer complement-targeting therapeutics that reach the choriocapillaris layer in effective concentrations at earlier disease stages.

In summary, we demonstrate that BrM is a critical barrier to the movement of complement proteins in the eye, which has implications for the understanding of AMD pathogenesis and the design of therapeutic approaches. BrM separates complement regulation in the choroid and retina, but certain key molecules can cross BrM. In particular, complement activation in the choriocapillaris will release C5a, which can then traverse BrM and initiate responses in the RPE resulting in inflammation and angiogenesis.

## Ethics Statement

Our study uses tissue from human donor eyes, where human eyes were obtained from the Manchester Royal Eye Hospital Eye Bank after removal of the corneas for transplantation. Our research adhered to the tenets of the Declaration of Helsinki. In all cases, there was prior consent for the eye tissue to be used for research, and guidelines established in the Human Tissue Act of 2004 (UK) were followed. Ethical approval for the use of human donor eyes was given by the University of Manchester Local Research Ethics Committee 3 (ref. 11305).

## Author Contributions

SC lead the project, performed all western blots, diffusion, and gel analysis experiments and wrote the manuscript. VT expressed recombinant FHL-1 proteins and NB and SM collected, phenotyped, and processed human donor eyes. PB contributed to experimental design and writing of the manuscript.

## Conflict of Interest Statement

SC and PB are inventors on the patent GB1709222.2. All other authors declare no conflict of interest.
