# Supplementary material for: Bruch’s Membrane Compartmentalizes Complement Regulation in the Eye with Implications for Therapeutic Design in Age-Related Macular Degeneration
Source: Front Immunol. 2017 Dec 19;8:1778. doi: 10.3389/fimmu.2017.01778 (PMC5742201; doi:10.3389/fimmu.2017.01778)
Supplement: Supplementary file 1 [file Data_Sheet_1.docx]

**
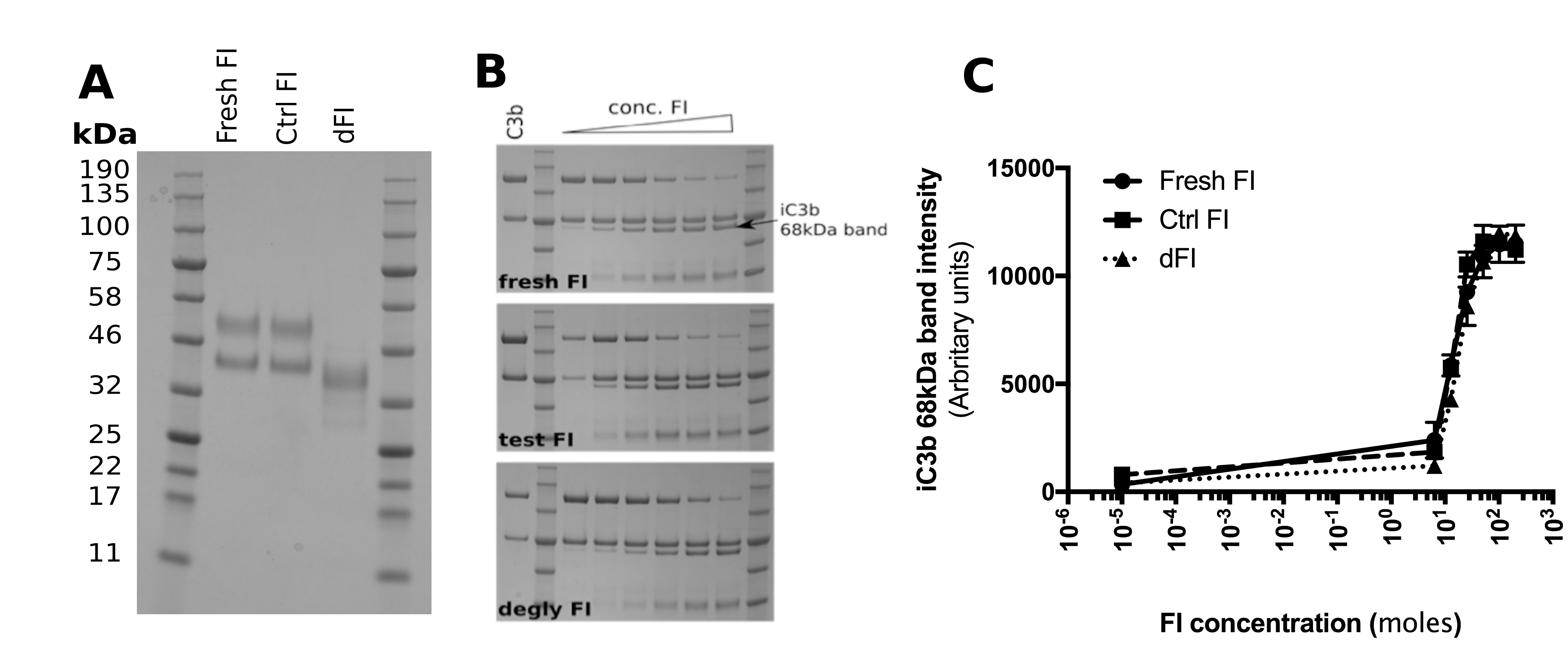
**

**Figure S1. Chemical deglycosylation of FI does not affect its C3b breakdown activity.** (**A**) SDS-PAGE gel showing the deglycosylation of native FI (FI) and the resulting band shift pattern: the heavy chain band is most glycosylated hence the greatest movement. (**B**) The non-reducing deglycosylation protocol resulted in deglycosylated FI that retains its enzymatic function, where the density of the iC3b 68kDa product can be measured and can be plotted against FI concentration (**C**).

**
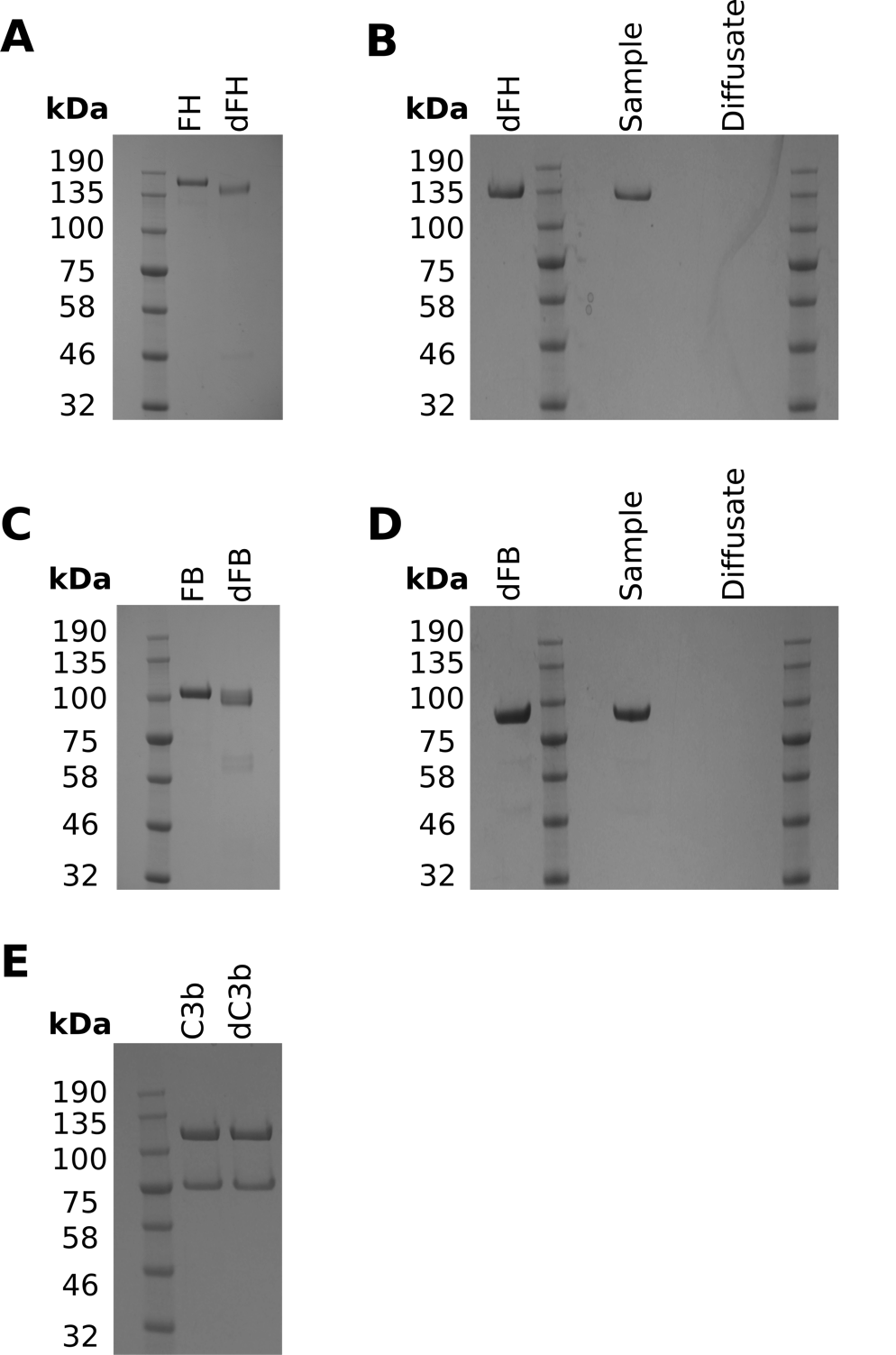
**

**Figure S2. Deglycosylation of complement proteins results in changes to gel band migration.** (**A**) Deglycosylation of FH results in the 155kDa band migration pattern shifting to an apparent molecular weight of 135kDa. (**B**) dFH is still unable to penetrate macula BrM in diffusion experiments. (**C**) A similar band migration change is observed with dFB, and this structural change also has no effect on the passage of dFB through macula BrM (**D**). (**E**) C3b was not susceptible to deglycosylation using the non-denaturing protocol in this study.
